# The Effects of a Non-Technical Skills Training Program on Emotional Intelligence and Resilience in Undergraduate Nursing Students

**DOI:** 10.3390/healthcare10050866

**Published:** 2022-05-07

**Authors:** Diana Jiménez-Rodríguez, María del Mar Molero Jurado, María del Carmen Pérez-Fuentes, Oscar Arrogante, Nieves Fátima Oropesa-Ruiz, José Jesús Gázquez-Linares

**Affiliations:** 1 Department of Nursing, Physiotherapy and Medicine, University of Almeria, 04120 Almería, Spain; 2 Department of Psychology, Faculty of Psychology, University of Almeria, 04120 Almería, Spain; mmj130@ual.es (M.d.M.M.J.); mpf421@ual.es (M.d.C.P.-F.); foropesa@ual.es (N.F.O.-R.); 3 Red Cross University College of Nursing, Spanish Red Cross, Autonomous University of Madrid, 28003 Madrid, Spain; oscar.arrogante@cruzroja.es; 4 Department of Psychology, Universidad Autónoma de Chile, Providencia 7500000, Chile; jlinares@ual.es

**Keywords:** emotional intelligence, nursing students, psychoeducational course, resilience, university training

## Abstract

There is a growing body of research on emotional intelligence and resilience in nursing students. However, there is little evidence of the development of these variables in intervention programs. This study aims to analyze the effects of a non-technical skills training program in emotional intelligence and resilience. Sixty students in the second year of Nursing Education from a Spanish public university completed this psychoeducational course. The pre-experimental design was longitudinal with pre- and post-intervention evaluation. The course consisted of 12 sessions of classroom education, three small-group workshops and a set of individual activities. Results in the emotional intelligence dimensions showed that Clarity (t = 3.10, *p* = 0.003) and Repair (t = 3.59, *p* < 0.001) increased significantly after participation in the program. Furthermore, the participants had a higher Resilience index when they had completed the program, with a statistically significant difference from the pre-course mean (t = 2.83, *p* = 0.006). This non-technical skills training program was an effective method of improving emotional intelligence and resilience in nursing students. Therefore, its use is recommended as a psychoeducational strategy for training undergraduate nursing students, as it improves their personal and professional competencies, resulting in higher-quality care.

## 1. Introduction

Promoting, establishing and maintaining health is a very great challenge in the nursing profession. These professionals hold an influential position in relation to the benefits derived from health, which demands strong dedication to their practice, performed in close proximity to patients and their families. In severe or more complicated patient care situations, nurses are exposed to a major risk of stress [1], which can negatively affect their own health and professional practice. Emotional intelligence and the capacity for resilience are personal skills that improve occupational health and make it possible to cope more effectively with the challenges of clinical nursing practice [2], and therefore, some authors propose its development during their university training and later in professional practice [3,4,5,6,7]. Resilience has also been considered a variable that influences stress [8]. The main objective of this study was to test the effect of non-technical skills training in a psychoeducational intervention program for nursing students.

Emotional intelligence enables complex processing of one’s emotions, guiding thought and action [9]. According to the Four Branches Model formulated by Mayer and Salovey [9], four specific interrelated domains intervene in emotional competence: Perceiving emotions, emotional facilitation of thinking, understanding emotions and emotion regulation. Perceiving emotions refers to the perception, identification, appraisal and expression of emotions, both one’s own and of others. Emotional facilitation of thinking establishes a two-way relationship, in which emotions have a relevant role in facilitating thinking. Emotional understanding involves analyzing emotions, knowing how to recognize them and label them verbally, as well as interpret them within a given context. Finally, emotional regulation involves recognizing one’s own and others’ emotional states, from observation and emotional distancing, where emotions must be modified, without losing their meaning.

People with high emotional intelligence have more confidence in themselves, are more optimistic and understand others better, which enables them to cope more successfully with environmental demands and pressures [10,11]. In the nursing profession, emotional intelligence has been related to job satisfaction, job commitment, stress reduction and protection from burnout [11,12,13,14,15,16,17,18].

Emotional intelligence has also been associated with positive results of nursing students. Studies agree that high scores on emotional intelligence in samples of nursing students are related to better adjustment to university life [19,20] and performance in curricular practice [21]. Some studies have found positive correlations of emotional intelligence and academic performance in nursing education [3,22], while in others emotional intelligence scores were not associated with performance [23]. Although in some studies, nursing students showed higher emotional intelligence scores than students in other degree programs [7,24], a large number of researchers around the world have suggested that training in emotional intelligence should be part of a nursing degree course, because it significantly benefits their individual, professional and social wellbeing [3,4,25], favoring effective decision-making, contributing to stress reduction in situations of major difficulty in clinical practice [2,20,26] and facilitating the transition to the role of nursing [27].

Development of resilience is important for mitigating stress related to continual exposure of nursing professionals to the emotional demands of relations with patients and their families and with other healthcare professionals [28]. Resilience is the ability to come out strengthened in spite of adversities. It is a personality characteristic that facilitates people’s adaptation to hostile circumstances, reducing the harmful effects of stress and enabling successful development [29,30]. According to Wagnild and Young [30] the capacity for resilience can be defined based on two main dimensions: Personal competence and acceptance of oneself and of life. Personal competence is measured in self-confidence, independence, decision, ingenuity and perseverance. Acceptance of oneself and of life implies adaptability, balance, flexibility and a stable perspective of life. Studies have found a relationship between emotional intelligence and resilience in the area of health [6,31,32], in which high scores in resilience are associated with higher scores in emotional intelligence. Resilience of nursing students has also been related to preparation for and satisfaction with clinical practice [33,34].

However, there are very few empirical studies on the effectiveness of emotional intelligence training programs for nursing students or professionals [4,5], and those that study development of the capacity for resilience are practically nonexistent. Foster et al. [35] identified a variety of strategies for improving students’ emotional intelligence skills and recommended a skills-based model for curricula and learning and teaching approaches. Some of the studies analyzing the effects of intervention programs on emotional intelligence are the following: Lee and Gu [5], developed and evaluated the effects of a 20-h emotional intelligence development program, carried out in eight sessions over four weeks, with a sample of 36 nursing students. The quantitative results showed that emotional intelligence, communication skills, the capacity for resilience, stress coping strategies and clinical competence were significantly higher in the experimental group than in the control. According to the qualitative results, after participating in the emotional intelligence program, the nursing students experienced an improvement in emotional intelligence, interpersonal relations and empowerment, as well as a reduction in the stress associated with clinical practice. Kozlowski et al. [4] performed a study measuring emotional intelligence of 60 nurses before and three months after participating in an emotional intelligence program. Intervention consisted of a five-hour workshop on emotion management in oneself and others, one 30-min individual feedback session and an individualized follow-up reminder sent by SMS. The results showed a significant increase in emotional intelligence scores in the group that received the training, while the scores in the control group did not increase. Other interventions, focused on improving communication skills, personal growth, leadership or self-reflection in nursing, showed an improvement in emotional intelligence after the program [35,36,37,38,39].

In view of the results of the research above, the objective of this study consisted of evaluating the efficacy of a non-technical skills training program on emotional intelligence and resilience for nursing students. In contrast to the quoted earlier similar intervention studies, our training program combines both individual and group interventions aimed at a better understanding and management of emotions. In addition, our time intervention is longer than previous studies, improving an adequate non-technical skills development that needs more time and experience for their control. Lastly, we have provided a psychologically safe environment to nursing students during the training program, providing them with a shared belief that the team is safe for interpersonal risk-taking. Our main hypothesis was that emotional intelligence and resilience of university students would improve after participation in the program.

## 2. Materials and Methods

### 2.1. Study Design

This pre-experimental descriptive study had a longitudinal design with a single-group pre- and post-intervention evaluation. The research and reporting methodology followed STROBE-cross-sectional studies.

### 2.2. Setting and Sample

The participants in this study were second-year undergraduate nursing students at the University of Almeria, a public university in Spain. A non-technical skills training program was completely integrated into the “Health Promotion and Safety” course in the second-year study plan for a Degree in Nursing. Participation in the study was offered to all the students and their acceptance was voluntary. A total of 129 students were enrolled in this course. At first, the sample was 111 students. Of these, 60 students completed the psychoeducational program in which non-technical skills training was included and answered the questionnaire afterwards. The study was carried out from October 2019 to January 2020.

### 2.3. Procedure

The non-technical skills training program consisted of a theory and practice course taking up matters related to emotions, emotion management and resilience as part of the second-year subject “Health Promotion and Safety”. The teaching strategies employed were general knowledge sessions on the subject of study with explanatory videos and case-solving; small-group work (18 students) with experiencing and role-playing. Finally, individual activities related to promoting resilience were scheduled to be done at home.

The program was implemented by an instructor trained in teaching emotional intelligence and resilience. Table 1 shows the program undertaken.

The students filled out a self-report questionnaire at the beginning (pretest) and at the end of the course (posttest).

### 2.4. Data Collection Instruments

First, an ad hoc questionnaire was applied to collect sociodemographic and educational data (age, sex, previous healthcare training, and so forth), as well as a series of items for assessing the attitudes and evaluation of including work in non-technical skills in the practice training program. This was answered on a five-choice Likert-type response scale (1 = Totally disagree, 2 = Disagree, 3 = Neither agree nor disagree, 4 = Agree, 5 = Totally agree), where the participants could express how much they agreed with the items. Each item was written with little variations to adjust to the pre- and post-intervention measures. Specifically, they were asked about the importance of working on non-technical skills (PRE “I think it is important to work on non-technical skills in degree coursework”; POST “I think working on emotions in this course can help me in future care practice”), evaluation of the weight of nontechnical skills in the undergraduate curriculum (PRE “I think more hours should be devoted to degree training in the skills we are indicating”; POST “I think more hours should be devoted to degree training in the skills we are indicating”) and finally on the need to promote/widen these contents in the training planned for the degree (PRE “I think it is appropriate to include non-technical skills in Nursing Degree education”, POST “I would like training in emotional skills to be included throughout the degree coursework”).

On the one hand, to evaluate the emotional intelligence we used the Trait Meta-Mood Scale (TMMS-24) by Fernández-Berrocal et al. [40] based on the Trait Meta-Mood Scale (TMMS) by Salovey et al. [41]. It is a self-report measure consisting of expressing one’s opinion of one’s own emotional abilities and skills on a five-point Likert-type scale, from 1 = strongly disagree to 5 = strongly agree. The TMMS-24 evaluates three dimensions of emotional intelligence in eight items for each component: Emotional attention, Clarity and Repair. The scale has optimum psychometric properties in different samples [40,42]. Emotional attention refers to the level of awareness of one’s own feelings and moods (e.g., “I pay much attention to my feelings”), Clarity is the ability to understand one’s own moods (e.g., “I am usually very clear about my feelings”) and Repair refers to the capacity for regulating feelings and emotional states (e.g., “Although I sometimes feel sad, I usually have a mostly optimistic outlook”). In this study, internal consistency was high for all the subscales: ω = 0.88 and GLB = 0.94 on attention, ω = 0.89 and GLB = 0.96 in Clarity, and ω = 0.86 and GLB = 0.94 in Repair.

On the other hand, to evaluate the resilience the Resilience Scale (RS) was used. This was a brief version validated by Wagnild [43] based on the original 25-item resilience scale [30]. In this study, the Spanish adaptation was used. This abbreviated version has 14 items which were rated on a seven-point Likert-type scale, from 1 = strongly disagree to 7 = strongly agree. The scale measures two dimensions of resilience: Personal competence (e.g., “I usually manage one way or another”) and Acceptance of oneself and of life (e.g., “I usually take things in stride”). Higher scores on the scale show more resilience. The scale shows adequate psychometric properties in different university student samples [44,45,46]. In this study, the internal consistency index for the whole scale was optimum at ω = 0.91 and GLB = 0.92.

### 2.5. Data Analysis

First, frequency analyses were done of the students’ attitudes and opinions on practice programs that include non-technical training content as taken from their answers at the two measurement times (before and after program startup). To check the relationship between the study variables (emotional intelligence and resilience), bivariate correlation analyses were done for the two measurement times (pre and post). When the correlations had a *p*-value near 0.05, the Vovk-Sellke maximum *p*-ratio or the maximum diagnosticity of a two-sided *p*-value was computed [47,48].

The Student’s t for related samples was found to check for any variations in the emotional intelligence and resilience scores before and after participation in the program. The effect size was estimated by Cohen’s d [49] based on the difference in mean scores.

To examine the reliability of the instruments used for data collection, McDonald’s Omega coefficient was estimated, following the proposal and indications of Ventura-León and Caycho [50]. The Greatest Lower Bound (GLB) was also calculated.

### 2.6. Ethical Considerations

The study was approved by the Research Ethics Committee from the Department of Nursing, Physical Therapy, and Medicine from the University of Almeria (n° EFM-26/19). All the participants were informed of the study objectives and gave their written consent for participating in the study. Research followed the recommendations of the Helsinki Declaration.

## 3. Results

### 3.1. Participants’ Characteristics

The mean age of the sample was 20.60 (SD = 4.92). The sex distribution of the sample was 73.3% (*n* = 44) women and 26.7% (*n* = 16) men, with mean ages of 20.91 (SD = 5.65) and 19.75 (SD = 1.69), respectively. Their prior healthcare training was: 80% had no training, and of the remaining percentage, 8.3% (*n* = 5) were certified nursing assistants, 5% (*n* = 3) were laboratory technicians, 3.3% (*n* = 2) both (nursing assistant and laboratory technician), 1.7% (*n* = 1) X-ray technician, and the 1.7% (*n* = 1) remaining had another university degree (Physiotherapy).

### 3.2. Non-Technical Skills Training during the Course: Attitudes and Evaluation

First, concerning the importance of studying non-technical skills in their degree coursework, before the program, 18.3% (*n* = 11) of the sample agreed and 81.7% (*n* = 49) strongly agreed with the importance of working on these skills. After participation in the program, 25% (*n* = 15) of the students agreed on the usefulness of the program in future care practice, and 71.7% (*n* = 43) strongly agreed.

Concerning the perceived need for improving their non-technical skills, before participation in the program, most of them said they agreed (33.3%, *n* = 20) or strongly agreed (63.3%, *n* = 20) with it. After the program, 53.3% (*n* = 32) agreed and 40% (*n* = 24) strongly agreed with the need to continue working in this line.

Concerning their opinion of the hours of degree education devoted to non-technical skills, 36.7% (*n* = 22) agreed and 55% (*n* = 33) strongly agreed that they were insufficient. After their participation in the program, 35% (*n* = 21) agreed and 56.7% (*n* = 34) strongly agreed, that more time should be devoted to this type of training for the degree.

Finally, most of the students had positive attitudes in their evaluation of non-technical content in the nursing degree, both before participating in the program (40% in agreement and 58.3% strongly in agreement), and afterwards (38.3% agreed and 58.3% strongly agreed).

### 3.3. Effects of the Non-Technical Skills Training Program in Emotional Intelligence and Resilience

First, the results of the correlations found (pre and post), between the emotional intelligence dimensions and resilience are shown in Table 2.

**Figure 1:**
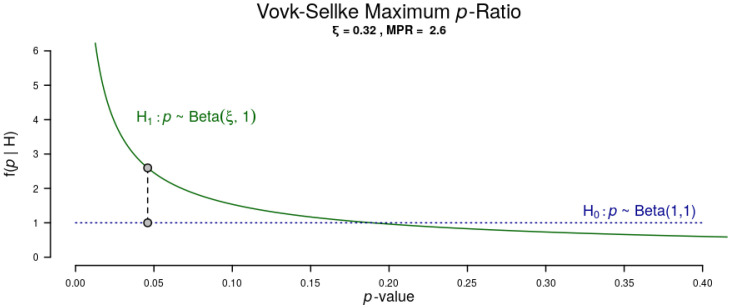
Vovk-Sellke maximum *p*-ratio (Emotional attention) (Note. Based on a two-sided *p*-value, the maximum possible odds in favor of H_1_ over H_0_ = 1/(−e *p* log(*p*)) for *p* ≤ 0.37 [46]).

The positive relationship between the two dimensions of emotional intelligence (Clarity and Repair) and Resilience is maintained in both measurements (pre and post). Furthermore, the correlation between Emotional attention and Resilience was negative before participation in the program began, and after it ended, that correlation between the two variables was lost. The level of significance of this relationship, before the program was near 0.05 (*p* = 0.046), so its diagnostic value was tested. Figure 1 shows the Vovk-Sellke maximum *p*-ratio: the maximum diagnosticity of a two-sided *p*-value [47,48]. In the Emotional attention–Resilience correlation (before participation in the program), a *p* = 0.046 would lead us to reject the null hypothesis, however, it is only 2.6 times more probable under the best H_1_ than under H_0_.

**Figure 2:**
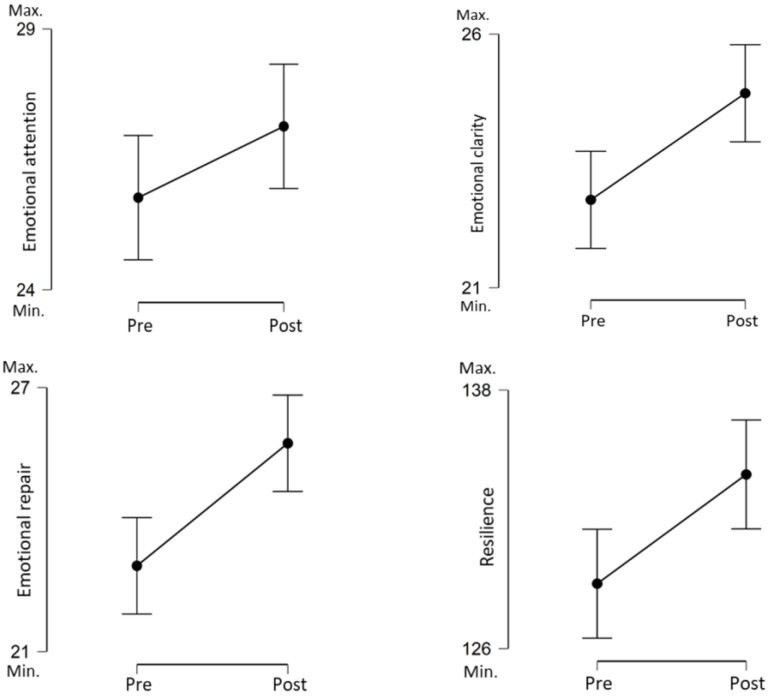
Emotional intelligence and resilience. Descriptive plots.

The statistically significant differences between the measurements before and after the non-technical skills training program are shown in Table 3, and graphically in Figure 2.

The scores in the emotional intelligence dimensions, Clarity and Repair, increased significantly after participation in the program. Nevertheless, in Emotional attention, the differences between measurements were tendential. Participant Resilience was statistically significantly higher than the pre-program mean after completing the program.

## 4. Discussion

This study evaluated the efficacy of a non-technical skills training program in topics related to emotions, emotion management and resilience for undergraduate nursing students in Spain. There are not many studies analyzing these behaviors in intervention programs over time. The main results of the study have shown that after participation in the program, the students scored higher in the Emotional Intelligence dimensions of Clarity of feelings and Emotional repair. Similar results have been found in other studies evaluating emotional intelligence in university student samples, although with other measurement instruments [4,5]. Some authors suggest that training in emotional intelligence in nursing improves problem-solving and perceived competence, and therefore, ensures better-quality performance and less stress and facilitates decision-making [1,20]. In our study, no significant differences were found in Emotional attention of the students at the end of the program. Emotional attention is the ability to express and experience feelings appropriately. Perhaps the training in Emotional Attention required more sessions with self-reflection and personal growth activities to help develop students’ self-esteem. Further information on this idea may be found in the studies by Foster et al. [35], Pai [36] and Park and Lee [37].

According to the Four Branches Model formulated by Mayer and Salovey [9], our psychoeducational training program improved the four domains included in this model, but more statistically significantly the emotional competencies related to emotion perception and understanding (Clarity dimension of emotional intelligence), and emotion regulation (Repair dimension of emotional intelligence). In this sense, our nursing students improved their ability to understand their moods, perceiving adequately their emotions and analyzing them, knowing how to recognize them and label them verbally, as well as interpret them within a given context. Moreover, our nursing students improved their capacity for regulating feelings and emotional states, recognizing one’s own and others’ emotional states, from observation and emotional distancing, where emotions must be modified, without losing their meaning. Although our nursing students did not statistically significantly improve their emotional facilitation of thinking according to the Four Branches Model, it is recommended to increase the sample size in future studies.

However, scores were statistically significantly above the pre-program scores on capacity for resilience at program completion. Intervention for resilience is important, as it can facilitate nurses’ resources for dealing with the effects of emotional dissonance in their work [28], enabling them to come out strengthened from difficult situations and increases their optimism. Moreover, this capacity must be considered a mediating variable in the relationship between emotional intelligence and performance in nursing [6].

The results of this study show that inclusion of a psychoeducational program in undergraduate nursing education is highly recommendable, as it would contribute to improving their personal and professional competencies, leading to high-quality care. Based on these findings, our study suggests the need to include training programs in non-technical skills in emotional intelligence and resilience in nursing education study plans to train students in coping more successfully with the challenges of clinical practice.

Nonetheless, the evaluation of the effects of this program is based on short-term results, and it would be advisable to evaluate these variables over a longer period of time. Such measurements would enable decisions to be made on widening training in such non-technical skills to higher courses. Furthermore, future research could have an experimental design including a control group to be able to compare the results. Finally, these findings, are from a program for Spanish university students, and therefore, adaptation to other contexts are suggested so results can be generalized.

## 5. Conclusions

Intervention programs on emotional intelligence and resilience for nurses are few at this time. The success of this non-technical skills training program could inspire other nursing schools to set up similar programs and contribute to the development of these personal competencies. Keeping in mind the complexity of the patient care environment, professors of nursing education who prepare students to promote health, should consider a focus on non-technical skills along with training in technical competencies.

Therefore, the university environment may be an ideal context for increasing students’ emotional intelligence and resilience. The study plan of future nursing professionals should be directed and recognizing and valuing training in emotional intelligence and resilience by starting up effective programs that prepare the future nursing professionals to cope successfully with clinical practice.

## Figures and Tables

**Table 1:** Non-technical skills training program.

	Classroom Sessions	Small-Group Workshops	Individual Activities
Timetable	Weekly 1.5-h subject teaching sessions (12 weeks. Total: 18 h).	1.5-h workshops	30 days of individual reflective work
Objective	Formation in emotions and resilience	Self-exploration and emotion management	Promote resilience
Work methodology	Theory: -Basic principles of emotion management and resilience Practice: -Presentation of cases and solutions -Group discussion and reflection on audiovisual material	Experiential exercises directed at self-knowledge, questioning emotions and emotion management. Later group reflection. -Workshop 1: Rage and fear. -Workshop 2: Sadness. -Workshop 3: Approach to real situations in role-play to improve emotion management	-Review and put into practice personal self-care activities -Focus on the positive: observe positive things during the day for 21 days. -Learn to grow with problems

**Table 2:** Emotional intelligence and resilience. Pearson correlations and descriptive plots.

	Pre		Post	
	Pearson’s r	95% CI	Pearson’s r	95% CI
Emotional attention–Resilience	−0.25 ^1^	(−0.481, −0.005)	0.01	(−0.242, 0.266)
Emotional clarity–Resilience	0.51 ^2^	(0.295, 0.677)	0.63 ^2^	(0.458, 0.768)
Emotional repair–Resilience	0.58 ^2^	(0.389, 0.731)	0.69 ^2^	(0.540, 0.809)

^1^ *p* < 0.05; ^2^ *p* < 0.001.

**Table 3:** Emotional intelligence and resilience. Paired samples Student’s *t*-test.

	Measures				Pairs Post-Pre						
	Pre		Post		t	*p*	MD ^3^	SE ^4^	95% CI		Cohen’s d
	M ^1^	SD ^2^	M ^1^	SD ^2^					Lower	Upper	
Attention	25.76	6.21	27.13	5.80	1.62	0.110	1.36	0.84	−0.31	3.05	0.21
Clarity	22.73	5.72	24.83	6.05	3.10	0.003	2.10	0.67	0.74	3.45	0.40
Repair	22.95	5.98	25.73	6.81	3.59	<0.001	2.78	0.77	1.23	4.33	0.46
Resilience	129.01	18.61	134.08	17.13	2.83	0.006	5.06	1.78	1.49	8.64	0.36

^1^ M: mean score; ^2^ SD: standard deviation; ^3^ MD: Mean difference; ^4^ SE: standard error difference.

## Data Availability

The data presented in this study are available on request from the corresponding author.
